# Leisure time physical activity and the risk of hip or knee replacement due to primary osteoarthritis: a population based cohort study (The HUNT Study)

**DOI:** 10.1186/s12891-016-0937-7

**Published:** 2016-02-16

**Authors:** Marianne Bakke Johnsen, Alf Inge Hellevik, Valborg Baste, Ove Furnes, Arnulf Langhammer, Gunnar Flugsrud, Lars Nordsletten, John Anker Zwart, Kjersti Storheim

**Affiliations:** Communication and Research Unit for Musculoskeletal Disorders, Oslo University Hospital, Oslo, Norway; Faculty of Medicine, University of Oslo, Oslo, Norway; Department of Orthopaedic Surgery, Oslo University Hospital, Oslo, Norway; The HUNT Research Centre, Department of Public Health and General Practice, Faculty of Medicine, Norwegian University of Science and Technology (NTNU), Levanger, Norway; Uni Research Health, Bergen, Norway; Register, Department of Orthopaedic Surgery, Haukeland University Hospital, Bergen, Norway; Departments of Clinical Medicine, University of Bergen, Bergen, Norway

**Keywords:** Physical activity, Osteoarthritis, Joint replacement, Hip, Knee, Risk factors

## Abstract

**Background:**

The relationship between leisure time physical activity (LPA) and hip and knee OA and subsequent joint replacement has not yet been clearly defined. Some studies have found the risk of knee replacement (TKR) to increase with high levels of LPA, while others have found no overall relationship to either TKR or hip replacement (THR). The aim was to investigate the association between LPA and the risk of severe end-stage OA, defined as THR or TKR due to primary OA, in a large population-based cohort.

**Methods:**

Participants in the Nord-Trøndelag Health Study (HUNT) were followed prospectively to identify THR and TKR using the Norwegian Arthroplasty Register. Self-reported LPA was classified as inactive, low, moderate or high. The Cox proportional hazards model was used to calculate hazard ratios (HRs) according to levels of LPA with adjustments for confounding variables. Analyses were performed by age (<45, 45–59 and ≥60 years) and sex.

**Results:**

A total of 66 964 participants (mean age 46.8 years (SD 16.3) were included in the analyses. We identified 1636 THRs and 1016 TKRs due to primary OA during 17.0 years (median) of follow-up. High LPA was significantly associated with THR for women <45 years (HR 1.78, 95 % CI 1.08–2.94) and men between 45–59 years (HR 1.53, 95 % CI 1.10–2.13) at baseline. A significant trend was found only among women < 45 years at baseline (p = 0.02). We found that LPA was significantly associated with TKR for women only (HR 1.45, 95 % CI 1.03–2.04). No measures of LPA were associated with TKR for men.

**Conclusion:**

In this population-based study, high level of LPA was associated with increased risk of THR where a significant trend of LPA was seen among women <45 years at baseline. For TKR, high LPA was associated with increased risk only in women. In contrast to previous studies, this study shows a possible association between high LPA and the risk of THR.

**Electronic supplementary material:**

The online version of this article (doi:10.1186/s12891-016-0937-7) contains supplementary material, which is available to authorized users.

## Background

Osteoarthritis (OA) is ranked the 11^th^ highest contributor to global disability with the hip and knee representing the highest proportion of the OA burden [1]. Joint replacement is a proxy outcome measure for severe, symptomatic end-stage OA [2]. The frequency of these procedures is rising in Western countries [3, 4]. In total, 12 920 hip and knee replacements were performed in Norway in 2013, which represented a 20 % increase from 2008 [5]. The lifetime risk of symptomatic OA is also high and has been estimated to 25 % and 45 % for hip and knee, respectively [6, 7]. The high prevalence of OA, its social costs, and its impact on physical function and quality of life underlines the importance of identifying factors influencing its management and prevention. Known risk factors for hip and knee OA include older age, obesity, heavy, physical workload, joint injury and physical activity [8–10]. However, the relationship between leisure time physical activity (LPA) and the development of hip and knee OA [10, 11] or future joint replacement [12–15] has not yet been clearly defined. Previous cohort studies have found the risk for total knee replacement (TKR) to increase with high levels of LPA [12, 15], while others have found no overall relationship to either TKR or total hip replacement (THR) [13, 16]. Intensive exercise and sport participation have more consistently showed an increased risk of later THR and TKR in former athletes [14, 17]. The inconsistent results from previous studies may to a large extent be related to differences in study design, source population, definition of LPA and definition of OA. Few prospective studies have in the same population compared the risk of THR or TKR in relation to LPA as the main exposure of interest [12, 15]. The main objective of the present study was, therefore, to investigate the incidence and association between levels of LPA and the risk of severe OA, defined as THR or TKR, due to primary OA in a large population-based cohort.

## Methods

### Study population

**Fig. 1:**
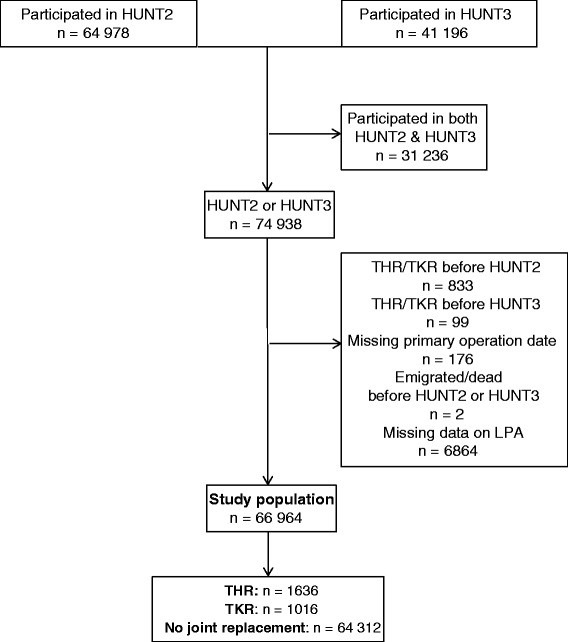
Flow chart of the study population. HUNT = The Nord-Trøndelag Health Study, HUNT2 (1995-97) and HUNT3 (2006-08), THR = total hip replacement, TKR = total knee replacement, LPA = leisure time physical activity

We used data from the Nord-Trøndelag Health Study (HUNT), a large population-based cohort covering 125 000 Norwegian participants. The HUNT Study collected data in three surveys; HUNT1 (1984-86), HUNT2 (1995-97) and HUNT3 (2006-08). Data collection is described in detail elsewhere [18]. All inhabitants aged ≥ 20 years living in the county of Nord-Trøndelag, Norway, were invited to participate through an invitation letter sent by mail. The HUNT Study includes data from questionnaires, interviews, clinical measurements and biological samples. For the purpose of the present study, we included 74 938 persons with data from questionnaires and clinical measurements in HUNT2 (n = 64 978) and new participants in HUNT3 (n = 9960). A total of 1110 participants were excluded because of a joint replacement prior to baseline based on information from the Norwegian Arthroplasty Register (n = 932), because no date had been recorded for the primary joint replacement (n = 176) or because they had died or emigrated before start of follow-up (n = 2). Data on death and emigration was obtained from HUNT, which receives the information from Statistics Norway. In addition, 6864 individuals were excluded because of missing data on LPA. The study population is, therefore, comprised of 66 964 participants (Fig. 1).

### Exposure of interest

Information about LPA was collected in the standard questionnaire by asking “How much of your leisure time have you been physically active in the last year?” Commute to and from work counted as leisure time. Participants were asked to report their average weekly hours of light LPA (no sweating or being out of breath) and/or hard LPA (sweating or being out of breath) with four response alternatives (0, <1, 1–2, ≥3 h) for each intensity level. The two questions of light and hard LPA were not mutually exclusive. For the purpose of the current study, a LPA variable based on the above categories of light and hard LPA was constructed [19]. Participants were categorized into the following four groups: inactive (no light or hard LPA), low (<3 h of light and no hard LPA), moderate (≥3 h of light and/or <1 h hard LPA) and high (≥1 h hard and any light LPA). As the ‘inactive group’ represented a very small number of participants, ‘low LPA’ was used as a stable reference group in the regression analyses. Validation of the LPA questions in HUNT showed that especially hard LPA correlated well with VO_2max_ and the International Physical Activity Questionnaire (IPAQ) and had moderate test-retest reliability [20].

### Covariates

Potential confounders included workload, which was collected in the questionnaire by asking “How would you describe your work (including both paid and unpaid employment)?” with four mutually exclusive response options: 1) mostly sedentary work (office or assembly work), 2) work that requires walking (teaching, shop assistant, light industrial work), 3) work that demands walking and lifting (postman, nurse, construction work) or 4) heavy physical work (heavy construction work, farming). Smoking was categorized as never, past or current smoker. Alcohol consumption during the last two weeks was divided into four categories: 0, 1–4, 5–14 and >15 units [21]. Diabetes, myocardial infarction, angina pectoris and stroke/brain hemorrhage were defined by affirmative answers to the questions: “Have you had or do you have any of the following: diabetes and/or myocardial infarction (MI) and/or angina and stroke?” Cardiovascular disease (CVD) was defined as a composite of MI, angina or stroke [22]. No distinction was made between diabetes type 1 and type 2. Height and weight were measured by trained personnel. Body mass index (BMI) is weight in kilograms divided by height in meters squared.

### Outcome

The study end-points were primary THR or TKR as indicators for severe end-stage OA. The unique 11-digit identity numbers of Norwegian citizens enabled us to link individuals’ data in HUNT with the corresponding joint replacement data in the Norwegian Arthroplasty Register (NAR). The orthopaedic surgeon submits a standardized form for each joint replacement performed containing information on indication for and type of THR or TKR. We included only joint replacements due to primary OA, which is defined in NAR as a joint replacement caused by idiopathic OA. All participants with THR or TKR as a result of previous injury (e.g sequelae after ligament and menisci injuries) were censored. In addition, joint replacements secondary to rheumatoid arthritis, sequelae after femoral neck fracture, congenital dysplasia, Perthes’ disease/epiphysioloysis, ankylosing spondylitis and osteonecrosis of the femoral head were censored. Patients with more than one joint replacement (hip or knee) were only counted once. NAR was started in 1987 by the Norwegian Orthopedic Association and was fully operational from 1989 for THRs [23] and 1994 for TKRs [24]. The completeness of registration in NAR has been reported at 96.5 % and 95.3 % for primary THRs and TKRs, respectively [5].

### Statistical analyses

Descriptive statistics are given as means and standard deviation (SD) or numbers and percentage. Follow-up was calculated from baseline in HUNT2 (1995-97) or HUNT3 (2006-08) to date of first THR or TKR or date of censoring. Participants were censored at date of first primary joint replacement from indications other than primary OA, date of death, date of emigration or end of follow-up (December 31, 2013), whichever came first. Crude incidence rates for joint replacements were given per 10 000 person-years. The Cox proportional hazards model was used to estimate the hazard ratios (HRs) and 95 % confidence intervals (CIs) for primary THR or TKR by levels of LPA.

Potential confounders, categorized as described, were included in a multivariable model. Adjustments for comorbidity (CVD and diabetes) and lifestyle factors (smoking and alcohol use) did not affect the magnitude or direction of the estimates of LPA. Neither were the comorbidity and lifestyle factors significant in the multivariable model. Thus, our final (full) model included age at baseline, sex, BMI and workload in addition to LPA. BMI was used as a continuous variable. Age showed to be a strong confounder in our analyses. To further investigate the effect of age, we performed stratified analyses according to sex and age at baseline (<45, 45–59 and ≥60 years). Age was in addition controlled for as a continuous variable within each age group.

For testing trends, the LPA variable was included as a pseudo-continuous variable in the regression model where the numbers for each LPA category (e.g. inactive = 0, low = 1) were treated as continuous numbers. To test whether the association between LPA and THR or TKR was modified by age or sex, models with and without interaction terms were compared using the likelihood ratio test. We performed sensitivity analyses to assess the robustness of our findings by excluding those with self-reported OA at baseline. The OA diagnosis was confirmed in the questionnaire by affirmative answer to the question “Has a doctor ever said that you have degenerative joint disease (OA)?” However, no information was obtained about the joint(s) affected. Test and visual inspection of plotted scaled Schoenfeld residuals showed that the proportional hazard assumption was satisfied for all variables, except for age which was stratified on. Values of p < 0.05 were considered to be statistically significant. All statistical analyses were performed using SPSS version 21 (SPSS Inc., Chicago, IL) and Stata 13.0/IC (StataCorp LP, College Station, TX, USA). The current study was approved by the Regional Committee for Medical Research Ethics (2013/151/REK Sør-Øst C). The HUNT Studies and the NAR have been approved by the REK and the Data Inspectorate of Norway. All participants in the HUNT Study and the NAR have signed a written informed consent, including linkage with other registries. The manuscript reporting adheres to STROBE guidelines for reporting observational research (Additional file 1).

## Results

A total of 31 825 men and 35 139 women were included in the analyses. We identified 2652 (4.0 %) joint replacements (1636 THRs and 1016 TKRs) due to primary OA during 959 706 person-years (median 17.0 years, maximum 18.4 years) of follow-up. A total of 554 participants were censored because of joint replacements for conditions other than primary OA. Participants with high LPA were younger at baseline and at a younger age when they received a joint replacement compared to those less active. More men than women had a high level of LPA (Table 1).

**Table 1:** Baseline characteristics of the study population by levels of leisure time physical activity

	Leisure time physical activity			
	Inactive (n = 4914)	Low (n = 19 611)	Moderate (n = 22 419)	High (n = 20 020)
Age in HUNT, years (mean, SD)	55.1 ± 18.8	50.3 ± 15.8	47.2 ± 16.1	40.8 ± 14.3
Age in NAR, years (mean, SD)	71.1 ± 8..9	68.9 ± 8.9	69.5 ± 9.4	66.3 ± 9.7
Time from baseline to final follow-up, years (mean, SD)	13.1 ± 5.8	14.5 ± 4.9	14.5 ± 4.9	14.3 ± 5.0
BMI, kg/m^2^ (mean, SD)	27.3 ± 4.9	26.7 ± 4.3	26.2 ± 4.0	25.7 ± 3.7
Sex, n (%)				
-Women	2503 (50.9)	11 706 (59.7)	11 978 (53.4)	8952 (44.7)
-Men	2411 (49.1)	7905 (40.3)	10 441 (46.6)	11 068 (55.3)
Workload, n (%)				
-Sedentary	1035 (21.1)	4685 (23.9)	5643 (25.2)	5566 (27.8)
-Walking	764 (15.6)	4913 (25.1)	6058 (27.0)	5166 (25.8)
-Walking & lifting	739 (15.0)	4006 (20.4)	4682 (20.9)	4548 (22.7)
-Heavy physical work	672 (13.7)	1454 (7.4)	1698 (7.6)	2423 (12.1)
-Missing	1704 (34.7)	4553 (23.2)	4338 (19.4)	2317 (11.6)
Smoking, n (%)				
-Never	1791 (36.5)	7643 (39.0)	9388 (41.9)	10 003 (50.0)
-Previous	1274 (25.9)	5359 (27.3)	6245 (27.9)	5099 (25.5)
-Current	1748 (35.6)	6306 (32.2)	6496 (29.0)	4642 (23.2)
-Missing	101 (2.1)	303 (1.6)	290 (1.3)	276 (1.4)
Alcohol consumption, units/2wk, n (%)				
- 0	2245 (45.7)	6901 (35.2)	6612 (29.5)	4167 (20.8)
- 1-4	1643 (33.4)	8410 (42.9)	9645 (43.0)	8690 (43.4)
- 5-14	760 (15.5)	3694 (18.8)	5254 (23.4)	5989 (29.9)
- >15	202 (4.1)	445 (2.3)	742 (3.3)	1027 (5.1)
-Missing	64 (1.3)	161 (0.8)	166 (0.7)	147 (0.7)
CVD, n (%)	703 (14.3)	1486 (7.6)	1384 (6.2)	614 (3.1)
-Missing	9 (0.2)	44 (0.2)	43 (0.2)	17 (0.1)
Diabetes, n (%)	271 (5.5)	613 (3.1)	533 (2.4)	309 (1.5)
-Missing	8 (0.2)	34 (0.2)	28 (0.1)	14 (0.1)

*HUNT* The Nord-Trøndelag Health Study, *NAR* Norwegian Arthroplasty Register, *BMI* body mass index, *2wk* consumption over a 2-week period, *CVD* cardiovascular disease

### THRs due to primary OA

The crude rate indicated a decreased incidence of THRs by increasing level of LPA. The incidence of THR was higher in women than men (Table 2). In the age-adjusted analyses there was a significant association between LPA and THR for men in high LPA (HR 1.34, 95 % CI 1.08–1.66). We found similar results after adjusting for BMI and workload in addition to age (full model). The full model, stratified by age and sex, revealed a significant association between high LPA and THR for women < 45 years (HR 1.78, 95 % CI 1.08–2.94) and men between 45–59 years at baseline (HR 1.53, 95 % CI 1.10–2.13). A significant trend (p = 0.02) of increased risk of THR by increasing level of LPA was apparent among women <45 years at baseline (Table 2).

**Table 2:** Incidence and risk of hip replacement (THR) in relation to leisure time physical activity (LPA)

						Model 1^a^			
				Incidence rate	Age-adjusted	Total population	<45 years	45-59 years	≥60 years
LPA	No-of subjects	Person-years	No. of THRs	per 10 000 person-years	HR (95 % CI)	HR (95 % CI)	HR (95 % CI)	HR (95 % CI)	HR (95 % CI)
Women									
Inactive	2503	31 373	98	31.2	0.90 (0.72–1.12)	0.80 (0.59–1.10)	0.76 (0.23–2.52)	1.02 (0.63–1.67)	1.09 (0.71–1.68)
Low	11 706	173 034	419	24.2	1	1	1	1	1
Moderate	11 978	175 911	358	20.4	0.98 (0.85–1.13)	0.99 (0.83–1.17)	1.06 (0.63–1.78)	1.00 (0.79–1.27)	1.04 (0.78–1.40)
High	8952	125 019	159	12.7	1.02 (0.84–1.23)	1.04 (0.84–1.29)	1.78 (1.08–2.94)	0.98 (0.73–1.30)	1.02 (0.67–1.57)
p trend					0.55	0.31	0.02	0.86	0.96
Men									
Inactive	2411	32 978	52	15.8	1.24 (0.90–1.70)	1.25 (0.87–1.79)	0.74 (0.16–3.52)	1.49 (0.90–2.46)	1.24 (0.72–2.13)
Low	7905	111 566	147	13.2	1	1	1	1	1
Moderate	10 441	148 919	205	13.8	1.08 (0.88–1.34)	1.16 (0.91–1.47)	1.62 (0.70–3.75)	1.10 (0.77–1.58)	1.24 (0.86–1.79)
High	11 068	160 906	198	12.3	1.34 (1.08–1.66)	1.33 (1.04–1.69)	1.83 (0.81–4.14)	1.53 (1.10–2.13)	1.06 (0.71–1.60)
p trend					0.07	0.11	0.08	0.10	1.00

^a^Model 1: adjusted for age at baseline, BMI and workload

### TKRs due to primary OA

Corresponding to the results of THR, higher incidence of TKR was seen among women compared to men and the crude indicated a decreased incidence of TKR by increasing level of LPA (Table 3). In the age-adjusted analyses there was a negative association between moderate LPA and TKR for women (HR 0.80, 95 % CI 0.66–0.96) (Table 3). After adjusting for BMI and workload in addition to age (full model), high LPA showed a borderline significant increased risk of TKR for women (HR 1.29, 95 % CI 1.00–1.67). The full model, stratified by age and sex, revealed that this association was strongest for women between 45–59 years at baseline (HR 1.45, 95 % CI 1.03–2.04). LPA was not associated with the risk of TKR among men (Table 3).

**Table 3:** Incidence and risk of knee replacement (TKR) in relation to leisure time physical activity (LPA)

						Model 1^a^			
				Incidence rate	Age-adjusted	Total population	<45 years	45–59 years	≥60 years
LPA	No-of subjects	Person-years	No.of THRs	per 10 000 person-years	HR (95 % CI)	HR (95 % CI)	HR (95 % CI)	HR (95 % CI)	HR (95 % CI)
Women									
Inactive	2503	31 373	65	20.7	0.99 (0.75–1.30)	0.84 (0.58–1.22)	0.69 (0.16–2.93)	0.77 (0.40–1.49)	1.20 (0.73–1.99)
Low	11 706	173 034	270	15.6	1	1	1	1	1
Moderate	11 978	175 911	190	10.8	0.80 (0.66–0.96)	0.87 (0.70–1.10)	0.94 (0.51–1.74)	0.81 (0.59–1.12)	1.02 (0.70–1.50)
High	8952	125 019	107	8.6	1.04 (0.82–1.31)	1.29 (1.00–1.67)	1.42 (0.78–2.61)	1.45 (1.03–2.04)	1.17 (0.70–1.98)
p trend					0.47	0.09	0.20	0.09	0.99
Men									
Inactive	2411	32 978	34	10.3	1.12 (0.77–1.65)	1.13 (0.74–1.72)	0.27 (0.04–2.11)	1.33 (0.76–2.34)	1.32 (0.65–2.65)
Low	7905	111 566	110	9.9	1	1	1	1	1
Moderate	10 441	148 919	135	9.1	0.95 (0.74–1.22)	1.08 (0.81–1.43)	1.06 (0.50–2.25)	1.22 (0.83–1.81)	0.99 (0.60–1.63)
High	11 068	160 906	105	6.5	0.88 (0.67–1.15)	0.96 (0.71–1.29)	0.99 (0.46–2.10)	1.07 (0.71–1.59)	0.85 (0.49–1.48)
p trend					0.19	0.57	0.44	0.83	0.30

^a^Model 1: adjusted for age at baseline, BMI and workload

There was no evidence for effect modification by age or sex on the associations between LPA and THR or TKR. The number of participants with self-reported OA at baseline were 5244 (8 %) of 66 964. After excluding participants with self-reported OA, the direction of risks were the same as in the main analyses; however, the magnitude of the associations between high LPA and THR (Additional file 2) or TKR (Additional file 3) were somewhat stronger. A significant trend (p = 0.03) of LPA for THR was apparent for men between 45–59 years at baseline.

## Discussion

In this population-based cohort study we found that LPA was associated with increased risk of THR due to primary OA. For TKR, LPA was associated with increased risk for women only. The effect was first and foremost related to high LPA, as no significant association was observed for less vigorous LPA. This is, to our knowledge, the first study to present the association between LPA and the risk of THR and TKR in relation to different age groups.

In contrast to previous population-based cohort studies, we found an association between high LPA and subsequent THR [12, 13, 15]. This suggests that mechanical load or abnormal stress induced by high LPA may affect the hip and knee in the same way, although the underlying anatomical structures differ. Our findings are however in line with studies that have found an increased risk of THR after sports participation [14, 17]. The high incidence of THR in our study gave us statistical power to detect any potential associations, while other studies may have been underpowered [13]. For TKR, we found an increased risk of related to high LPA for women only. Higher incidence [25] and prevalence [26, 27] of OA has been found in women as compared to men, especially after menopause. Inherent biological and hormonal differences offer a possible explanation for this variation between men and women. The incidence of both THR and TKR have also been reported to be higher in women than men in Norway [28, 29], which agrees with the results in our study.

In an Australian cohort study, high LPA was associated with increased risk of TKR (HR 1.46, 95 % CI 1.13–1.87), but not for THR [15]. However, they had no detailed information on occupational physical demands, which may have left residual confounding related to workload. The follow-up time in this study was in average 4.8 years (SD 0.7), which may have been somewhat short to follow the onset and progression of OA to joint replacement. As they propose, LPA may have different effects across the spectrum of disease from a healthy joint to severe end-stage OA [15]. By the short follow-up they might have limited the associations to the effects of LPA in those with already OA.

Two previous Norwegian cohort studies found no association between LPA and THR or TKR [13, 16]. However, in these two studies [13, 16], LPA were not the main exposure of interest. Their definition of high LPA were highly sports related, requiring participation in hard training or competitions regularly and several times a week. The authors state that the small number of participants and joint replacements in the most active category may partly be a reason for not finding any effect of LPA on THR or TKR [16].

Similarly, a Swedish cohort study found no consistent relationship between LPA and THR or TKR other than showing a protective role of high LPA for women on the risk of THR (HR 0.66, 95 % CI 0.48–0.89) [12]. They had no information on workload, which may have affected the direction and magnitude of association. Physical workload, in comparison to LPA, has consistently shown an increased risk of hip and knee OA for both men and women [30, 31]. We found a protective effect of moderate LPA among women on the risk of TKR in the age-adjusted analyses (HR 0.80, 95 % CI 0.66–0.96). However, this effect was attenuated and non-significant after adjusting for workload and BMI in the full model. It is possible, as proposed earlier [12], that absence of adequate measurement tools to quantify LPA leads us to misclassify LPA in relation to OA. Self-reported measures may not capture the type and true intensity of LPA, although if validated they are often the most feasible to use in large cohort studies.

The association we found between LPA and joint replacement was first and foremost present for high LPA. We cannot rule out the possibility that participants with high LPA included a subgroup of individuals who engaged in intensive exercise and sports, beyond recreational activities. Previous studies have shown that those who exercise more regularly or intensely have an increased risk of hip and knee OA, although the risk seems to be highly related to previous joint injury as well as being overweight [9, 32, 33]. Participation in specific sports, such as soccer, ice hockey, cross country skiing and team handball has shown to be associated with OA and later joint replacement [14, 17, 34]. A knee injury to the anterior cruciate ligament, especially with a combined injury to the menisci, has been shown to result in a significantly higher risk of radiographic knee OA [35]. Consequently, as we were interested in joint replacements due to primary OA, all joint replacements due to previous injury (e.g. sequela after ligament or menisci injury) and conditions other than primary OA were censored.

Michaelsson et al. [14] investigated a cohort of long-distance cross-country skiers and found that participation in multiple races and faster finishing time was associated with increased risk of THR or TKR. They proposed that we might overestimate the impact of high LPA if previous injury is not sufficiently accounted for because these two can act together. However, adjustment for previous disease and injury did not substantially change their results. Another study investigated the prevalence of hip and knee OA and subsequent joint replacement in former male athletes. They found an increased odds ratio after adjusting previous injury in addition to age, BMI and occupational load [17]. Thus, we may both over- and underestimate the effect of LPA by including injury into the analyses.

Age was a strong confounder in our analyses, which changed the direction and magnitude of risk. In the stratified analyses, we found no association of LPA and joint replacement for those at 60 years or older at baseline. With ageing come comorbidities which could act as contraindications for joint replacement, but also reduce the level of LPA. This is in line with previous studies in which LPA was found to have little effect on the risk of developing hip or knee OA in middle-aged and older people [11, 36]. In the sample from the Framingham Offspring cohort without knee OA at baseline, they found that recreational activities like walking or jogging for exercise neither protected nor decreased the risk of radiographic or symptomatic knee OA, regardless of being overweight or not [11]. Cheng et al. [36] found that high LPA was associated with OA of the hip and knee only in young men (age 20–49).

Similar results have been found in studies investigating BMI, where weight gain in early adulthood (between age 20–40 at screening) was associated with increased risk of both THR and TKR [37, 38]. The cumulative effect of excess bodyweight over several decades compared to those who gain weight later in life was proposed as one possible explanation for the increased risk of OA and later joint replacement [39]. This may offer a potential explanation for LPA as well, suggesting that exposure at certain age may be more important than at other ages [40]. Active individuals may assert the necessity for, and undergo, arthroplasty at a younger age to retain an active lifestyle both at work and in leisure time, and might be considered more eligible for surgery than older individuals due to less comorbidity. A delay of surgical intervention has shown to potentially have an impact on the success rate of surgery. Consequently, the number of operations performed in younger people has increased over the recent years [41].

### Strengths and limitations

The main strengths of our study include a large population-based cohort, data available from clinical measurements and questionnaires, long follow-up and a prospective design. However, we measured LPA at baseline only and had no information about change in LPA during follow-up or prior LPA that could have affected the association with joint replacement. As the LPA questions in HUNT did not include information on type of activity performed, we cannot say anything about the risk related to any specific activity or sport. Self-reporting LPA may be prone to overestimation compared to more objective measures of physical activity like accelerometers [42], at the same time lack of precision in measuring LPA may have biased the effects towards null.

We used joint replacement as a proxy for severe OA of the hip and knee. The limitation of this case definition is that only a small portion of the total OA population undergoes joint replacement, therefore we are not able to explain the full burden of hip and knee OA. The majority of hospitals in Norway are publicly funded and free of charge for the patient. Consequently, we do not think that socio-economic status represents a significant confounder to the provision of, nor the seeking of surgery. We excluded participants with joint replacement before baseline, but we cannot be certain that we detected all THRs and TKRs prior to HUNT2. This might be especially relevant for some of the older participants with a joint replacement before the register was fully operational for hip and knee in 1989 or 1994, respectively. Differential misclassification may have occurred if in older participants with joint replacements prior to baseline were distributed unequally across LPA-groups. However, the number of undetected joint replacements in our study period is expected to be insignificant.

We performed separate sensitivity analyses to investigate reversed causation. After excluding participants with self-reported OA at baseline, the effect of LPA on THR and TKR became somewhat stronger. This may suggest reversed causation, where subjects with OA are more likely to report low levels of LPA at baseline. Consequently, our main results may be considered as a conservative estimate, as we may have underestimated the true effect of LPA. We also have to acknowledge that multiple testing might have given significant findings that actually were due to chance. However, it was only for high LPA that we found an increased risk of joint replacement. This relationship was consistent both in the main analyses and sensitivity analyses.

We did not have information about previous injury, which has been a limitation in previous cohort studies as well [12, 15]. However, joint replacements secondary to injuries, e.g. ligament or menisci injuries, were censored in the analyses to protect us from misclassifying the diagnosis of the outcome (primary OA). If previous injury is not sufficiently accounted for, as mentioned, we might have over- or underestimated of the true risk of LPA. However, if LPA causes injury which subsequently results in OA and joint replacement, then injury is more likely a mediator and adjustments should not be made for the total effect of LPA.

Participants who were excluded from the analyses because of missing data on LPA were older, more often women, and had somewhat higher BMI and more comorbidities at baseline compared to those included in the analyses. A great proportion of participants who were missing data on LPA were also missing workload data (71 %). These findings are in line with previous non-participation studies from HUNT [43, 44]. Participants with LPA and workload data were in contrast younger, healthier and more often in work. Thus, our findings are first and foremost generalizable to similar study samples.

Even though our findings indicated higher risk of future joint replacement related to high LPA, there are undeniably beneficial effects of being physically active. Weight-bearing activities have beneficial effects for bone health, general health and mortality, and moderate to high intensity of bone-loading is recommended, including a combination of endurance training and resistance exercises [45, 46]. Exercise has also been shown to reduce pain and improve physical function in those already diagnosed with hip or knee OA [47, 48] and potentially reduce or delay the need for joint replacement [49]. However, we acknowledge the importance of identifying subgroups that are at a higher risk of injury and early development of OA in order to prevent the increasing demand of joint replacements and future burden of OA.

## Conclusion

In our large population-based study, we found that high LPA were associated with increased risk of THR due to primary OA, where a significant trend of LPA was seen among women <45 years at baseline. For TKR, high LPA was associated with increased risk only in women. There were no other relationship between LPA and increased risk of THR or TKR. In contrast to former studies, this study shows an association between high LPA and the risk of THR.

## Additional files

Additional file 1:
**The STROBE statement. Description of data: Checklist for reporting observational studies.** (PDF 147 kb) Additional file 2:
**Risk of hip replacement (THR) after excluding participants with OA at baseline (n = 5244).** Description of data: Results of separate sensitivity analyses. (PDF 298 kb) Additional file 3:
**Risk of hip replacement (THR) after excluding participants with OA at baseline (n = 5244).** Description of data: Results of separate sensitivity analyses. (PDF 298 kb)
