# Supplementary material for: Leisure time physical activity and the risk of hip or knee replacement due to primary osteoarthritis: a population based cohort study (The HUNT Study)
Source: BMC Musculoskelet Disord. 2016 Feb 16;17:86. doi: 10.1186/s12891-016-0937-7 (PMC4754866; doi:10.1186/s12891-016-0937-7)
Supplement: Additional file 2: — Risk of hip replacement (THR) after excluding participants with OA at baseline (n = 5244). Description of data: Results of separate sensitivity analyses. (PDF 298 kb) [file 12891_2016_937_MOESM2_ESM.pdf]

Additional file 1 Risk of hip replacement (THR) after excluding participants with OA at baseline (n=5244).

	Model 1*			
	Total population	<45 years	45-59 years	≥60 years
LPA	HR (95%CI)	HR (95%CI)	HR (95% CI)	HR (95% CI)
Women				
Inactive	0.81 (0.55-1.18)	0.85 (0.26-2.80)	1.05 (0.61-1.83)	0.99 (0.55-1.77)
Low	1	1	1	1
Moderate	0.99 (0.81-1.21)	1.06 (0.62-1.81)	1.04 (0.80-1.34)	1.03 (0.71-1.48)
High	1.11 (0.88-1.41)	**1.84 (1.10-3.08)**	0.96 (0.70-1.33)	1.27 (0.78-2.06)
p trend	0.20	**0.02**	0.86	0.44
Men				
Inactive	1.11 (0.72-1.71)	0.75 (0.16-3.54)	1.44 (0.81-2.55)	0.95 (0.45-2.00)
Low	1	1	1	1
Moderate	1.28 (0.97-1.68)	1.54 (0.66-3.60)	1.20 (0.81-1.76)	1.44 (0.93-2.23)
High	**1.45 (1.11-1.90)**	1.77 (0.78-4.02)	**1.68 (1.17-2.40)**	1.11 (0.68-1.81)
p trend	0.01	0.10	**0.03**	0.46

LPA= leisure time physical activity

*Model 1: adjusted for age at baseline, BMI and workload, including participants without OA at baseline and with non-missing values on LPA, n=61 720.
