# Supplementary material for: Leisure time physical activity and the risk of hip or knee replacement due to primary osteoarthritis: a population based cohort study (The HUNT Study)
Source: BMC Musculoskelet Disord. 2016 Feb 16;17:86. doi: 10.1186/s12891-016-0937-7 (PMC4754866; doi:10.1186/s12891-016-0937-7)
Supplement: Additional file 3: — Risk of hip replacement (THR) after excluding participants with OA at baseline (n = 5244). Description of data: Results of separate sensitivity analyses. (PDF 298 kb) [file 12891_2016_937_MOESM3_ESM.pdf]

Additional file 2 Risk of knee replacement (TKR) after excluding participants with OA at baseline (n=5244).

	Model 1*			
	Total population	<45 years	45-59 years	≥60 years
LPA	HR (95% CI)	HR (95%CI)	HR (95% CI)	HR (95% CI)
Women				
Inactive	0.59 (0.35-1.02)	0 (0)	0.75 (0.35-1.63)	0.85 (0.39-1.83)
Low	1	1	1	1
Moderate	**0.75 (0.57-0.98)**	0.85 (0.45-1.63)	0.80 (0.56-1.15)	0.70 (0.41-1.17)
High	1.32 (0.99-1.75)	1.40 (0.75-2.63)	**1.55 (1.06-2.26)**	1.15 (0.61-2.17)
p trend	0.05	0.09	0.06	0.98
Men				
Inactive	1.12 (0.69-1.81)	0.37 (0.05-2.96)	1.32 (0.72-2.44)	1.34 (0.56-3.21)
Low	1	1	1	1
Moderate	1.00 (0.72-1.39)	1.23 (0.53-2.85)	0.94 (0.61-1.47)	1.11 (0.60-2.03)
High	1.03 (0.74-1.44)	1.14 (0.49-2.66)	1.02 (0.66-1.57)	1.10 (0.58-2.09)
p trend	0.92	0.38	0.61	0.93

LPA= leisure time physical activity

*Model 1: adjusted for age at baseline, BMI and workload, including participants without OA at baseline and with non-missing values on LPA, n=61 720.
